# Utilization of a combined EEG/NIRS system to predict driver drowsiness

**DOI:** 10.1038/srep43933

**Published:** 2017-03-07

**Authors:** Thien Nguyen, Sangtae Ahn, Hyojung Jang, Sung Chan Jun, Jae Gwan Kim

**Affiliations:** 1 Gwangju Institute of Science and Technology, Department of Biomedical Science and Engineering, 123 Cheomdangwagi-ro, Buk-gu, Gwangju, 61005, Korea; 2 University of North Carolina at Chapel Hill, Department of Psychiatry, Chapel Hill, NC, 27599, USA; 3 Gwangju Institute of Science and Technology, School of Electrical Engineering and Computer Science, 123 Cheomdangwagi-ro, Buk-gu, Gwangju, 61005, Korea

## Abstract

The large number of automobile accidents due to driver drowsiness is a critical concern of many countries. To solve this problem, numerous methods of countermeasure have been proposed. However, the results were unsatisfactory due to inadequate accuracy of drowsiness detection. In this study, we introduce a new approach, a combination of EEG and NIRS, to detect driver drowsiness. EEG, EOG, ECG and NIRS signals have been measured during a simulated driving task, in which subjects underwent both awake and drowsy states. The blinking rate, eye closure, heart rate, alpha and beta band power were used to identify subject’s condition. Statistical tests were performed on EEG and NIRS signals to find the most informative parameters. Fisher’s linear discriminant analysis method was employed to classify awake and drowsy states. Time series analysis was used to predict drowsiness. The oxy-hemoglobin concentration change and the beta band power in the frontal lobe were found to differ the most between the two states. In addition, these two parameters correspond well to an awake to drowsy state transition. A sharp increase of the oxy-hemoglobin concentration change, together with a dramatic decrease of the beta band power, happened several seconds before the first eye closure.

In 2010, the foundation for traffic safety in US reported that 16.5% of fatal crashes occurring between 1999 and 2008 were related to driver drowsiness^1^. Drowsiness involves a decrease in the level of alertness. Although drowsiness can result from fatigue, they are different conditions. Fatigue is a cumulative process, which gradually impairs alertness, while drowsiness fluctuates rapidly over a period of several seconds^2^. Each fluctuation during drowsiness corresponds to a microsleep episode and is associated with the loss of attention^3^. During a microsleep episode, the lowered attention reduces the driver’s ability to judge and react to an unexpected situation and an accident is more likely to occur. In addition, Boyle *et al*. suggested microsleep episodes as a potential indicator of sleep onset^4^. Therefore, early identification of the microsleep episode could be helpful to detect the onset of driver drowsiness and consequently prevent automobile accidents.

Current methods for detecting driver drowsiness follow three directions, which are vehicle based performance monitoring^5^, driver behavior recording^6^ and driver physiological signal measuring^7^. Among them, the first two approaches are highly affected by external environments such as vehicle model and traffic condition, while the last one solely depends on subject condition; therefore, it shows a higher capability of detecting driver drowsiness. Measurement of the physiological signals includes neuronal electrical activity using EEG, eye movement using EOG, heart rate using ECG, muscle activity using EMG and tissue oxygenation using NIRS. Alteration of biosignals during drowsiness compared to normal condition has been examined in many studies. For instance, Patel *et al*. utilized ECG signal to show an increase in heart rate variability when mental workload decreases^8^. Slow eye movement in EOG data was found to be an indicator of drowsiness^9^. In addition, EEG has been widely applied and proven as a promising method for drowsiness studies^7^.

Among all modalities being used to measure brain signals, EEG is the most frequently used because of its high temporal resolution, portability and reasonable cost. Many advanced techniques have been developed to analyze EEG signals; including time domain analysis, frequency domain analysis, time-frequency analysis and a variety of classification techniques. The time domain analysis (time series analysis) is the most often chosen method because it provides the most important information that we want to measure. In addition, the time series analysis was proven to be more effective in processing EEG signals^10^,^11^. Due to the popularity of the EEG signal and its analysis methods, many works have been done to investigate drowsiness detection. In order to analyze the data, some studies focused on the analysis of frequency change between the awake and drowsy states^12^,^13^,^14^, while most other studies intended to classify the two states^13^,^15^,^16^,^17^,^18^,^19^. In our study, we used (1) frequency domain analysis to investigate the physiological signals in each state, (2) Fisher’s linear discriminant analysis (FLDA) to classify the awake and drowsy states, and (3) time series analysis to predict driver drowsiness.

In addition to EEG, NIRS was also employed to study driver drowsiness. Several studies have been conducted to investigate the hemodynamics response in the drowsy state^20^,^21^. Khan *et al*. attempted to classify the awake and drowsy states using NIRS signal^22^. In order to complement the information on hemodynamic response of NIRS to EEG, we utilized a combined EEG/NIRS system to study neuronal electrical activity and cerebral oxygenation change during the awake and drowsy states. The combination of these two modalities has been proven to be more efficient than each individual modality in brain computer interface applications^23^,^24^, seizures, epilepsy^25^,^26^,^27^,^28^, language studies^29^ and fatigue research^30^. Our study purpose is to investigate the potential of the combined EEG/NIRS system to detect drowsiness. In addition, we want to find the most informative parameters that can identify a drowsy state most accurately, such that we can minimize the signals used for a real time application.

## Results

### Subject’s state identification

**Figure 1:**
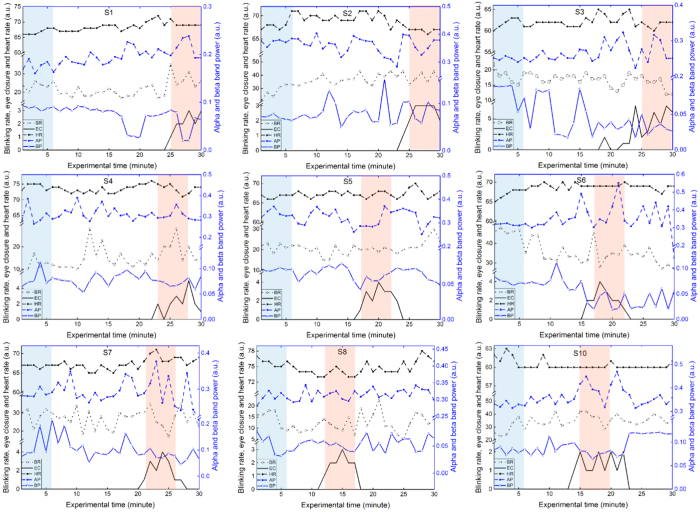
Subjects’ state identification using blinking rate (BR), eye closure (EC), heart rate (HR), alpha band power (AP) and beta band power (BP). The blue shaded region marks a 5 min-awake state and the red shaded region indicates a 5 min-drowsy state.

The blinking rate, eye closure, heart rate, alpha band power and beta band power of the nine subjects are shown (Fig. 1). All five parameters vary from time to time. However, with the exception of the beta band power, the variations of the eye-blinking rate, heart rate and alpha band power do not match with the variation of the eye closure. On the other hand, in most cases, the beta band power maintains a high value when the eye closure is zero and it decreases to a lower value when the eye closure increases. Hence, the identification of the subject’s state was primarily based on the beta band power and the eye closure. The awake state (5 min-awake, Fig. 1, blue shaded region) associates with a zero eye closure and high beta band power, while the drowsy state (5 min-drowsy, Fig. 1, red shaded region) corresponds to a positive eye closure and low beta band power. The nine subjects were in the awake state starting from the beginning of the experiment. However, the starting point of the drowsy state varied among subjects with the earliest time at 12 minutes and the latest time at 25 minutes after the experiment started.

### EEG band power in the awake and drowsy states

**Figure 2:**
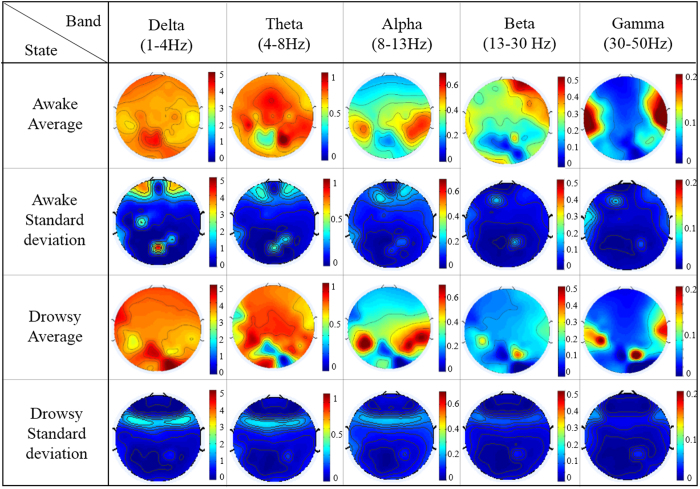
The grand-averaged and standard deviation topographies of the RPL values of five EEG bands of nine subjects in the awake and drowsy states.

Figure 2 shows the grand-averaged and standard deviation topographies of five band relative power level (RPL) of the nine subjects for 64 channels. Due to the diverse amplitude of each spectral band, different scales were applied. In the drowsy state, there is an increase in the RPL values in the lower frequency bands (delta, theta and alpha bands) and a decrease of the RPL values in the higher frequency bands (beta and gamma bands). The RPL values of the delta and theta bands are higher in most brain regions. The alpha band RPL values are higher in the parietal lobes. The beta band RPL values are lower in the frontal lobe and the gamma band RPL values are lower in both temporal lobes during the drowsy state. Except for the delta band power in the frontal cortex during the awake state, the low standard deviations in other regions, bands, and states indicate a small variation between subjects.

The results (p-values) from the unconnected statistical t-test are displayed in Table 1. All p-values are higher than the p-value of significance (0.002). However, the p-values from the frontal delta, the frontal and temporal beta and the central gamma are smallest among all obtained p-values. This implies that between the awake and drowsy states, there are some mild differences of the delta band in the frontal cortex, the beta band in the frontal and temporal cortex, and the gamma band in the central cortex, but not in the other bands and/or other brain regions.

### Hemodynamic response in the awake and drowsy states

The mean values of the oxy- and deoxy- hemoglobin concentration (HbO and Hb) changes were calculated for each subject in each state (Table 2). The average from all nine subjects results in a positive HbO change with a negative Hb change in the awake state and a negative HbO change with a positive Hb change in the drowsy state. The p-values of the t-tests for the HbO and Hb changes are included in the last line of Table 2. The low p-value (<0.05) indicates a significant difference of the HbO mean values between the awake and drowsy states. However, the p-value calculated from the Hb mean is higher than 0.05, which implies no statistical difference in the Hb change between two states.

### Classification accuracy

FLDA was employed to classify the awake and drowsy states using EEG alone, NIRS alone and combined EEG/NIRS. The classification accuracy is shown in Table 3. In most cases, when both EEG and NIRS signals were used together, the accuracy increases compared to the EEG alone (except for S3, S8, S10) or NIRS alone (except for S6, S8). The mean accuracy of the combined EEG/NIRS increases 8.7 percent compared to EEG alone and 5.5 percent compared to NIRS alone.

### Beta band RPL and HbO change during an awake to drowsy transition

**Figure 3:**
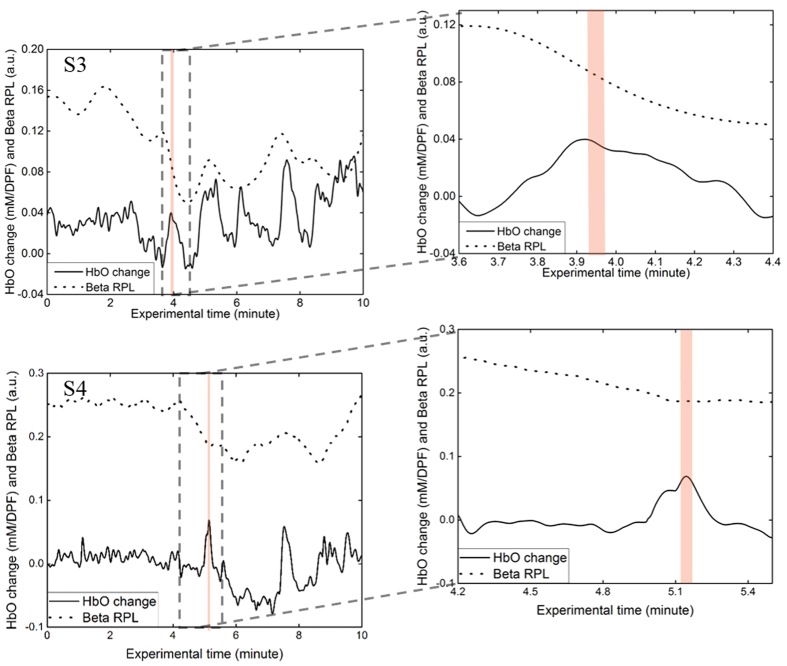
The time course of the beta RPL and the HbO change during an awake to drowsy state transition of S3 and S4. The red shaded regions indicate the first eye closure.

The representative data from S3 and S4 are displayed (Fig. 3). The red shaded regions in the graphs indicate the first eye closure (more than two seconds). Data from both S3 and S4 show a higher beta RPL and a relatively constant HbO change before the red shaded regions. However, between ten to fifty seconds before the red shaded regions, the beta RPL decreases and the HbO change increases dramatically. Data samples, including the red shaded regions, are enlarged in the right plots. A decrease in the beta RPL and an increase in the HbO change happens before the first eye closure. A similar trend was found in seven other subjects. This trend suggests the potential of these two parameters in the prediction of driver drowsiness.

### Drowsiness detection

For NIRS signals, the sharp increase of the HbO change during an awake to drowsy state transition (HbO-A-D) (Table 4, column 2) is used to predict drowsiness. Since during the awake state, the HbO was not constant but varied from time to time, the maximum variation of the HbO change during the awake state (HbO-A-max) is considered (Table 4, column 3). Based on the HbO-A-D and HbO-A-max of all subjects, a threshold of 0.05 is set. The subject is predicted to be in a drowsy state when the variation of the HbO change is greater than 0.05. The time difference between the HbO change-based predicted time and the first eye closure time (HbO-time) is shown in Table 4, column 4. With a threshold of 0.05, the HbO change can predict a drowsy state with 100% true positive (correctly detect drowsiness) and 22% false negative (misdetection of awake as drowsy state). In addition, the HbO change can predict the drowsy state earlier than the first eye closure with an averaged time of 4.3 seconds.

For EEG signals, the percentage change of the beta RPL during an awake to drowsy state transition (%Δ beta-A-D) (Table 4, column 5) is utilized to predict a drowsy state. Similar to the HbO change, the beta RPL exhibited some dramatic changes in the awake state. Hence, the maximum percentage change of the beta RPL during the awake state (%Δ beta-A-max) is taken into account (Table 4, column 6). Considering these two parameters, a threshold of 20% is set. The driver is predicted to be drowsy when the percentage change of the beta RPL is greater the threshold. The time difference between the beta RPL-based predicted time and the first eye closure (beta-time) is displayed in Table 4, column 7. On average, the beta RPL can predict the drowsy state 4.8 seconds earlier than the first eye closure. However, with the threshold of 20%, the beta RPL can predict the drowsy state with 100% true positive, but 78% false negative.

The investigation of the entire data set showed that the sharp decrease of the beta RPL together with the intense increase of the HbO change was unique for an awake to drowsy transition. During the awake state, a sharp decrease of the beta RPL sometimes happened and an intense increase of the HbO change occurred several times. However, these sudden changes did not happen at the same time point in the awake state. Hence, the combination of the beta RPL with the HbO change can decrease false negatives to 0% and keep true positives at 100% for the detection of the drowsy state.

### Drowsiness detection index

A drowsiness detection index (DDI), a binary variable, is derived to predict the drowsy state (equation (1)).


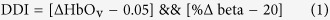


where, ΔHbO_v_ = HbO_c_ - HbO_l_, HbO_c_ is the current HbO change, HbO_l_ is HbO_c_ when HbO change decreases and HbO_l_ is the preceding lowest HbO change when the HbO change increase continuously. %Δ beta = (beta_h_ - beta_c_)/beta_h_, beta_c_ is the current beta RPL, beta_h_ is beta_c_ when the beta RPL increases and beta_h_ is the preceding highest beta RPL when the beta RPL decreases continuously.

**Figure 4:**
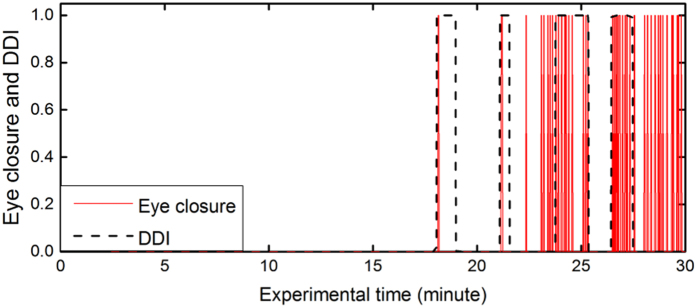
Eye closure and drowsiness detection index for S3 in 30 minutes.

The DDI is 1 when both (HbO_v_–0.05) and (%Δ beta–20) are positive. This means the drowsy state is predicted when the variation of the HbO change is greater than 0.05 and the percentage change of the beta RPL is higher than 20%. Figure 4 plots the eye closure and DDI during 30 minutes driving of S3. S3 first closed his eye at around 18 minutes after starting driving. The next eye closure was around 21 minutes. The DDI detects both times correctly, with four seconds earlier for the first time and three seconds earlier for the second time. Similarly, the DDI was capable of predicting eight other subjects’ drowsiness. The mean time of early prediction for nine subjects was 3.6 seconds, with a standard deviation of 1.4 seconds.

## Discussion

This study explored EEG and NIRS parameters that can indicate an awake to drowsy state transition. The neuronal electrical activity and the cerebral hemodynamic response were monitored simultaneously using EEG and NIRS in a simulated driving task, with subjects experienced both awake and drowsy states. The experimental results showed a good correspondence between the beta RPL and HbO change with the transition from the awake to drowsy state.

The increase of the lower frequency bands and the decrease of the higher frequency bands were previously observed for EEG in the drowsy state^12^,^13^,^14^,^15^,^16^,^17^,^18^,^19^. Studies of general drowsiness, which included the transition from awake to sleepy states, demonstrated an increase of the alpha rhythm^12^,^15^,^16^. Similarly, the change in the alpha activity was a common outcome found during drowsiness in both simulated and actual driving conditions^17^. In addition to the alpha rhythm, some researchers presented a decrease in the beta band^13^, while others claimed an increase in the delta and theta activities during low alertness level^14^,^18^,^19^. Similar to previous studies, our drowsy state EEG yielded a higher power in the delta, theta and alpha bands, and a lower power in the beta and gamma bands compared to the awake EEG data.

Instead of EEG, some studies used NIRS to monitor cerebral blood oxygenation during driving task. Li *et al*. found a significant reduction in the cerebral oxygen saturation when driver’s performance deteriorated^20^. Yoshino *et al*. showed no significant change in the cerebral oxygen exchange in the prefrontal cortex during constant velocity driving on an expressway^21^. Our study showed that in the prefrontal lobe, the HbO change had positive values and the Hb change had negative values in the awake state, while in the drowsy state, opposite changes were observed. In addition, several previous studies reported an increase of the HbO change in the frontal lobe at the beginning of the drowsy states^22^,^31^. In our case, the sharp increase of the HbO change was detected before the first eye closure. The increase of the HbO change can be due to the low oxygen consumption rate of the brain during the transition from an awake to drowsy state.

Even though we collected multimodal data of EEG, EOG, ECG and NIRS, we only used EEG and NIRS for further analysis because the variation of the blinking rate and heart rate did not correspond well with the subjects’ states. In addition, in order to approach an online drowsiness detection method, the signals from the frontal cortex should be targeted. From the statistical test, the delta and beta bands showed some mild differences between the awake and drowsy states among five EEG bands from the frontal lobe. However, during the awake state, the delta band exhibited high variation among subjects. In addition, the beta band power presented a high correspondence with the eye closure. Hence, the frontal beta band was selected as the most informative parameter for EEG. For NIRS, the HbO change was chosen as the most informative parameter because of its significant difference between the awake and drowsy states. For further study, we are now designing a small wireless EEG/NIRS system, with EEG and NIRS channels covering only the forehead.

The sharp increase of the HbO change and the dramatic decrease of the beta RPL was used to derive the DDI. Although the DDI can predict all subjects’ drowsiness correctly, it is still an ad-hoc index because of the chosen formula and thresholds. The thresholds for both beta RPL and HbO change could be altered depending on the subjects and systems used. In addition, with two parameters, the beta RPL and the HbO change, different approaches can be suggested to build an index for drowsiness detection. Therefore, in our study, the DDI was proposed as an example to illustrate the effectiveness of the utilization of the beta RPL and HbO change to predict drowsiness.

With a substantial increase of the classification accuracy between the awake and drowsy states when a combined EEG/NIRS was used, our study proved the usefulness of a multimodal system to study drowsiness. In addition, we found that the beta RPL from EEG and the HbO change from NIRS are the most informative parameters to predict drowsiness. Furthermore, a drowsiness detection index based on the distinct alteration of the beta RPL and HbO change before the first eye closure was proposed in this study.

However, there are still some limitations in this study. Firstly, we only did off-line analysis. Therefore, real-time data analysis needs to be developed in order to apply a combined EEG/NIRS system in actual driving conditions. Secondly, although we used both EEG and NIRS signals for the classification, the classification accuracy was not high enough (less than 80%). In the future, we should consider other EEG analysis techniques, such as complex network analysis^10^,^11^,^32^,^33^,^34^, to examine if we can get a better result. By using the complex network analysis, Gao *et al*. was able to obtain 100% accuracy in the classification of the healthy subjects and epilepsy patients^11^. Even though the small number of subjects (n = 9) in this study may limit our conclusion, the preliminary results demonstrated the capability of the combined EEG/NIRS system to predict the driver drowsiness in advance so that it can prevent motor vehicle collision caused by driver drowsiness.

## Materials and Methods

### Participants

Subjects were recruited from the students at the Gwangju Institute of Science and Technology (GIST). All procedures in the experiment were performed in accordance with the guidelines and regulations from the approval of the Institutional Review Board at GIST (20150615-HR-18-02-06). Eleven healthy subjects (S1 to S11, 1 female, aged from 24 to 28) agreed to participate in the study. All the subjects signed an informed consent agreement before the experiment. The night before the experiment, the subjects were asked to take enough sleep (at least seven hours) and not to consume any alcohol or caffeine-containing substances. When the subjects arrived for the experiment, the purpose of the study and experimental procedure were explained. After signing the document, the subjects were guided to practice driving to become familiar with the system. Before and after the measurements, the subjects were instructed to fill in a questionnaire to determine his/her alertness level.

### Experimental setup

**Figure 5:**
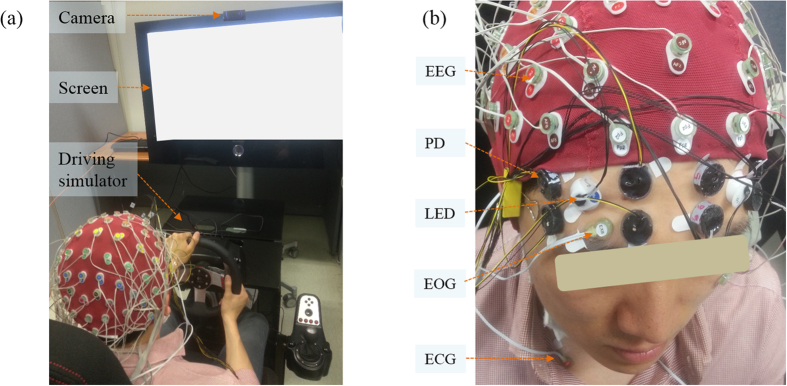
Experimental setup. (**a**) The simulated driving system and (**b**) the placement of EEG, EOG, ECG electrodes, and NIRS channels, which includes photodetector (PD) and LED.

The simulated driving system consisted of a racing wheel, including a brake and an accelerator (G27 Racing Wheel, Logitech, Switzerland) and a comfortable chair (GTS plus, PNS racing incorporated, South Korea) (Fig. 5a). A large screen was set up 1.5 m away from the chair. Commercial driving simulation software was used (Gran Turismo 5 on a PlayStation 3). The driving track had an oval shape with four curves, with two short and two long paths. The total time for a circuit was approximately three minutes with a velocity of 80 km/hour. The speed of the simulated system was limited to 100 km/hour.

A camera (HD Pro Web-Cam C920, Logitech, Switzerland) was attached on the top of the screen to record the subject’s behavior. An observer seated outside the experimental room monitored the subject’s behavior and decided the end time of the experiment. The experiment time ranged from 30–90 minutes depending on the subject’s condition. The shortest experiment time was 30 minutes, when a subject exhibited drowsy signs (more than two seconds eye closure and head nodding) during the first 30 minutes of driving. From the 30^th^ to 90^th^ minute, the experiment was ended ten minutes after the observer saw signs of drowsiness. In case a subject did not show any signs of drowsiness, the experiment was stopped after 90 minutes of measurement, and data from the subject were excluded from further analysis. During the experiment time, ten subjects (S1–S10) expressed the signs of drowsiness within the first 30 minutes of driving, while one subject (S11) stayed awake throughout 90 minutes of driving without eye closure or head nodding. In addition, S9 was sleepy at the beginning of the experiment. Therefore, for further analysis, we used the data from nine subjects (S1–S8 and S10) and rejected the data from S9 and S11.

Electrophysiological data were recorded using a Biosemi Active Two system with 64 EEG electrodes covering the head, 2 EOG electrodes attached near to the eyes and 2 ECG electrodes placed above the chest (Fig. 5b). The system acquired the signal using BCI 2000 software^35^ sampled at a rate of 512 Hz. The NIRS data, including oxy-, deoxy-, and total- hemoglobin concentration (HbO, Hb, and THb) changes, were collected in the prefrontal area, at a rate of 10 Hz, using a custom-built, 8 channel NIRS system (Fig. 5b). The detailed description of the NIRS system design and multimodal system synchronization can be found in an earlier work^30^.

### Data analysis

#### Data preprocessing

The acquired data were passed through a notch filter (60 Hz) to remove power line noise. After that, the signals were visually inspected. The EEG and NIRS channels, which contained abnormal noise, were excluded from analysis. Thereafter, EEG data were band-pass filtered from 1 Hz to 50 Hz, and NIRS data were low-pass filtered at 0.2 Hz. Then, independent component analysis (ICA) was applied to the EEG signal to decompose it to sixty-four ICA components. Each ICA component was manually checked. The components that contained artifacts such as eye blinking and movement artifacts, were selected to reject later. After rejecting bad components, the remaining components were used to reform a processed EEG signal. ECG and EOG signal were detrended to remove noise interference.

#### EEG relative power level and hemodynamic response

The preprocessed EEG data were decomposed into five frequency bands, delta (1–4 Hz), theta (4–8 Hz), alpha (8–13 Hz), beta (13–30 Hz) and gamma (30–50 Hz) bands. The power spectral density of a band was computed. Subsequently, RPL was calculated by dividing a band power with the summation of the power from five bands to reduce the variability between subjects^36^,^37^,^38^.

The NIRS modality used in our experiment was a continuous wave system. Therefore, the hemodynamic responses found were the relative changes of the HbO, Hb, and THb compared to a baseline. The baseline was set to be the first 10-second of data of each measurement. While the HbO and Hb changes were converted from the detected light intensity directly, the THb change was derived as the summation of the HbO and Hb changes. Hence, for further analysis, only the HbO and Hb changes were considered. In addition, due to the similar response from the eight frontal NIRS channels, the HbO and Hb changes were averaged over the eight channels.

#### Subject’s state identification

Driver drowsiness has been found to be characterized by increased blinking rate, decreased heart rate with more than two seconds of eye closure, increased alpha band power in the occipital lobe and decreased beta band power in the frontal lobe^2^,^20^,^21^,^22^,^24^. Hence, in our experiment, blinking rate, eye closure (more than two seconds), heart rate, alpha band power in the occipital lobe and beta band power in the frontal lobe were used to identify the subject’s current state. Eye blinking rate was derived from the EOG signal and heart rate was calculated by counting the number of ECG peaks. The recorded video was used to count the number of times a subject closed his/her eyes for more than two seconds. Alpha band power was averaged through 10 occipital channels and beta band power was averaged through 17 frontal channels. The five parameters were computed for each minute during thirty minutes of measurement. A condition of higher blinking rate^2^ with more than two seconds of eye closure, lower heart rate^2^, higher alpha band power^20^,^21^,^22^ and lower beta band power^24^ was considered to be a drowsy state. On the other hand, the condition with constant or opposite parameters was identified as the awake state. Five-minute data sections of EEG and NIRS in the awake state (5 min-awake) and in the drowsy state (5 min-drowsy) were selected for further analysis.

#### Statistical test

In order to find the most informative parameters that can be used to detect a drowsy state, we applied a simple, two-tailed student statistical test (t-test) to the hemodynamic response and five EEG bands’ power between the awake and drowsy states. For hemodynamic response, t-test was performed on the mean values of the HbO and Hb changes of the nine subjects. For EEG band power, the test was conducted for each band power in each brain region. Five brain regions, frontal (17 EEG channels), central (14 channels), temporal (6 channels), parietal (17 channels), and occipital (10 channels) regions, were defined using the EEG electrode placement map from Biosemi Active Two system.

#### Subject’s state classification

The frontal beta band RPL and the HbO change were used as the feature vectors for EEG and NIRS, respectively. The five-minute EEG and NIRS data were divided into 60 trials, each containing 5 seconds of data, for each state (awake and drowsiness). Data from the 120 trials were divided into 10 groups of 12 trials each. Among the 10 groups, 7 groups were randomly chosen for training and 3 groups were randomly chosen for testing. After then, each feature vector of training and testing data was fed into the classifier. The classifier, based on FLDA, was made using training group data, and testing groups were used as input to the FLDA classifier to generate classification accuracy. This procedure was repeated 120 times by choosing 7 training groups randomly thus 120 classification accuracies were estimated to obtain an average of classification accuracy. To investigate the combined effect of the classification using EEG and NIRS data, a combination of modalities with respect to classifiers’ outputs were performed. For the combination of classifiers in each modality, each classifier’s outputs (EEG and NIRS) were regarded as inputs of the next classifier. Thereafter, the outputs of the next classifier yield the results of the combined classifiers.

#### Detection of an awake to drowsy state transition

An awake to drowsy state transition was identified by the first eye closure. Based on the recorded video, the time point that a subject first closed his/her eyes for more than two seconds was marked. Ten-minute data samples, including the marked time, were extracted for further analysis. The beta band RPL and HbO change during the ten-minute data samples were investigated. The overall EEG and beta band power were calculated with a moving window of two seconds and one second overlap. Hence, the EEG power had a temporal resolution of 1 Hz. The beta band RPL was then computed by dividing the beta band power to the overall EEG power. The HbO change was down sampled to 1 Hz to have the same temporal resolution as the beta band RPL.

## Additional Information

**How to cite this article:** Nguyen, T. *et al*. Utilization of a combined EEG/NIRS system to predict driver drowsiness. *Sci. Rep.* **7**, 43933; doi: 10.1038/srep43933 (2017).

**Publisher's note:** Springer Nature remains neutral with regard to jurisdictional claims in published maps and institutional affiliations.

## Figures and Tables

**Table 1:** The p-value from the unconnected statistical t-test of each band in each brain region between two states.

Brain regions	Frontal	Central	Temporal	Parietal	Occipital
EEG band					
Delta	0.06	0.20	0.80	0.48	0.86
Theta	0.52	0.66	0.88	0.50	0.38
Alpha	0.44	0.66	0.24	0.16	0.34
Beta	0.10	0.20	0.10	0.38	0.22
Gamma	0.14	0.08	0.60	0.80	0.22

**Table 2:** Mean values of the HbO and Hb concentration changes from nine subjects in the awake and drowsy states and p-value from statistical t-test. DPF: differential pathlength factor.

Hemodynamic response	HbO (mM/DPF)		Hb (mM/DPF)	
Subject	*Awake*	*Drowsy*	*Awake*	*Drowsy*
S1	0.022	−0.002	−0.007	0.009
S2	0.073	−0.001	−0.024	0.016
S3	0.005	−0.017	−0.013	−0.017
S4	−0.011	−0.027	0.003	0.016
S5	0.017	−0.057	−0.009	−0.004
S6	0.036	−0.003	0.019	0.007
S7	0.022	−0.016	−0.011	0.009
S8	0.023	0.003	0.009	−0.001
S10	−0.013	−0.031	0.003	0.011
Mean	0.019 (±0.026)	−0.017 (±0.019)	−0.003 (±0.013)	0.005 (±0.010)
p-value	0.002		0.159	

**Table 3:** The classification accuracy using EEG alone, NIRS alone and combined EEG/NIRS for each subject and the mean values of the nine subjects.

Subject	S1	S2	S3	S4	S5	S6	S7	S8	S10	Mean
Modality										
NIRS	70.0	85.7	68.3	69.2	86.2	77.0	70.8	70.9	64.9	73.7 (±7.6)
EEG	72.4	62.2	90.4	56.8	71.0	63.0	67.1	77.9	73.4	70.5 (±9.9)
EEG/NIRS	74.5	99.5	84.1	78.6	86.4	72.6	74.5	70.5	71.7	79.2 (±9.4)

**Table 4:** Parameters of the HbO change and the beta RPL in the awake state and during an awake to drowsy state transition.

Subject	HbO -A-D	HbO -A-max	HbO -time	%Δ beta -A-D	%Δ beta -A-max	Beta - time
S1	0.09	0.03	5s	30	6	6s
S2	0.06	0.04	6s	24	26	3s
S3	0.08	0.01	4s	61	51	5s
S4	0.12	0.04	6s	33	35	4s
S5	0.13	0.05	5s	57	18	5s
S6	0.08	0.04	3s	27	23	5s
S7	0.16	0.10	5s	42	22	6s
S8	0.10	0.10	3s	33	33	4s
S10	0.06	0.03	2s	33	9	5s
Mean	0.10 (±0.03)	0.05 (±0.03)	4.3s (±2s)	38 (±13)	25 (±14)	4.8s (±1s)
